# Shape factor versus truncated cone‐based quantification of quadriceps and hamstring muscle volumes—A choice between accuracy and precision

**DOI:** 10.14814/phy2.70263

**Published:** 2025-03-25

**Authors:** Daniel P. Fitze, Nicola Mair‐Noack, Dominik Brun, Daniel Nanz, Jess G. Snedeker, Jörg Spörri

**Affiliations:** ^1^ Sports Medical Research Group, Department of Orthopaedics Balgrist University Hospital, University of Zurich Zurich Switzerland; ^2^ University Center for Prevention and Sports Medicine, Department of Orthopaedics Balgrist University Hospital, University of Zurich Zurich Switzerland; ^3^ Swiss Center for Musculoskeletal Imaging Balgrist Campus AG Zurich Switzerland; ^4^ Medical Faculty University of Zurich Zurich Switzerland; ^5^ Biomechanics Laboratory, Department of Orthopaedics Balgrist University Hospital, University of Zurich Zurich Switzerland; ^6^ Institute for Biomechanics ETH Zurich Zurich Switzerland

**Keywords:** hamstring muscles, magnetic resonance imaging, muscle volume, quadriceps muscles, segmentation

## Abstract

This study aimed to determine the average location of maximal anatomical cross‐sectional area of the quadriceps and hamstrings and to investigate the agreement of different muscle volume estimation methods. Magnetic resonance imaging datasets were acquired from 39 soccer players. Muscle volumes were calculated using slice‐by‐slice segmentation and compared with the shape factor and truncated cone‐based estimates. Descriptive data were expressed as means ± standard deviations, and Bland–Altman plots were used for agreement analyses. The average location of maximal anatomical cross‐sectional area was at 61 ± 10%, 64 ± 10%, 29 ± 3%, and 56 ± 6% for the vastus lateralis, rectus femoris, vastus medialis, and vastus intermedius, respectively. For the hamstrings, the maximal anatomical cross‐sectional area was at 45 ± 3%, 48 ± 9%, 58 ± 7%, and 38 ± 8% for the biceps femoris short head, biceps femoris long head, semitendinosus, and semimembranosus, respectively. Relative biases ranged from 2% to 9% for the shape factor and from 6% to 14% for the truncated cone method. The ranges of agreement were −15% to 29% and −1% to 19%, respectively. The shape factor method showed better accuracy, while the truncated cone method displayed better precision.

## 1 INTRODUCTION

Quantifying changes in muscle volume (MV) can provide valuable insights for sports and clinical practice. While MV may increase (i.e., muscle hypertrophy) during growth (Mersmann et al., 2017; O'Brien et al., 2010; Pitcher et al., 2012) or in response to resistance training (Hudelmaier et al., 2010; Maden‐Wilkinson et al., 2020; Roth et al., 2001), the consequences of traumatic injury (Lepley et al., 2019; Mühlenfeld et al., 2022; Psatha et al., 2012), musculoskeletal/neuromuscular disease (Beattie et al., 2012; Godi et al., 2016; Hart et al., 2012), an unloading environment (Alkner & Tesch, 2004; Marusic et al., 2021; Seynnes et al., 2008) or aging (Maden‐Wilkinson et al., 2013; Narici & Maganaris, 2007; Pinel et al., 2021) may include a decrease in MV (i.e., muscle atrophy).

Slice‐by‐slice segmentation of axial magnetic resonance imaging (MRI) slices is the current reference method for quantifying the volume of skeletal muscles (Pons et al., 2018). However, even semiautomated implementation of this method is very time‐consuming, which hinders regular application in sports and clinical practice, where test results need to be provided in a short period of time to plan and monitor training or rehabilitation phases. To overcome this issue, different MV estimation methods have been proposed and investigated in terms of time requirements, accuracy, and precision (Hudelmaier et al., 2010). These methods range from methods that estimate MV based on the muscle length, the maximal anatomical cross‐sectional area (ACSA_max_) and a muscle‐specific shape factor (Albracht et al., 2008; Domroes et al., 2021; Karamanidis et al., 2019; Mersmann et al., 2014, 2015) (i.e., the shape factor method) to methods that consider a reduced number of axial MRI slices for segmentation (Lund et al., 2002; Nordez et al., 2009; Tracy et al., 2003) (i.e., the truncated cone method).

The shape factor method was originally introduced by Albracht et al. (2008). It assumes that MV equals the product between muscle length, ACSA_max_, and a muscle‐specific shape factor, whereas the latter has a low coefficient of variation within a population. This estimation method has also been investigated in selected quadriceps muscles (Mersmann et al., 2015). In contrast, the truncated cone method requires multiple slices and interslice distances to calculate the MV from the sum of the subvolumes (i.e., from multiple truncated cone volumes). The truncated cone method is therefore often used when ultrasound (US) is the imaging modality of choice (Esformes et al., 2002; Franchi et al., 2020; Monti et al., 2020). However, for the shape factor method, it is essential to know the anatomical location of ACSA_max_ for a given muscle; otherwise, determining ACSA_max_ may undermine the simplicity and utility of the approach. Conversely, the accuracy of the truncated cone method clearly improves with an increasing number of segmented slices, but at the expense of time. Furthermore, it is not clear a priori which method might be best suited for different muscles and application contexts.

Therefore, the purposes of the present study were (1) to determine the average location of the ACSA_max_ relative to the muscle length for all quadriceps and hamstring muscles and (2) to quantify the agreement between two different in‐practice applicable MV estimation methods (i.e., the shape factor method and the truncated cone method) and the current reference method (i.e., slice‐by‐slice segmentation).

## 2 MATERIALS AND METHODS

### 2.1 Participants and ethics

Thirty nine female and male competitive soccer players (19 female: age = 21.8 ± 3.0 years, body height = 170.7 cm ± 8.3 cm, body mass = 67.1 kg ± 10.2 kg; 20 male: age = 21.6 ± 2.9 years, body height = 172.4 cm ± 9.0 cm, body mass = 68.4 kg ± 10.2 kg) were recruited for the present study. Recruitment was performed based on personal queries to soccer clubs in the respective leagues. Athletes aged between 17 and 35 years were included. A previous anterior cruciate ligament (ACL) tear and previous knee, ankle, or hip surgery were considered exclusion criteria. The underlying study protocol was approved by the Cantonal Ethics Committee Zurich (BASEC No. 2020–02583), and the study was conducted according to the ethical standards of the Declaration of Helsinki and national laws. All study participants signed a study‐specific informed consent form before participation.

### 2.2 Magnetic resonance imaging examination

MRI data were acquired with a 3‐Tesla system MAGNETOM Prisma (Siemens Healthcare GmbH, Erlangen, Germany). After safety screening, the study participants were positioned head first in a supine position on the patient table with the integrated 32‐channel spine coil and handed an emergency bell. They were instructed to position their arms laterally along the body. The legs were kept apart at hip‐joint width and in a slightly internally rotated position, stabilized by a tape that was applied around both feet. A crescent‐shaped pillow was placed under both knees, and two 18‐channel body‐array coils were positioned over the pelvis and thighs. Finally, in addition to the soft earplugs, the study participants were given headphones.

The imaging protocol included a fast‐view localizer, three fast axial proton‐density weighted turbospin‐echo scans at the upper ankle, knee, and hip joints for bone‐torsion evaluation, and a 4‐station spoiled 3D dual‐gradient echo VIBE DIXON sequence for muscle‐volume segmentation, followed by a variety of high‐resolution knee and hip joint scans.

At each station of the VIBE Dixon acquisition, 160 slices (slice thickness: 2.00 mm) were acquired with the following parameters: field‐of‐view: 460 mm and 332 mm in the readout (left/right) and phase‐encoded (anterior/posterior) directions, respectively; repetition time: 3.87 ms; first and second echo times: 1.23 and 2.46 ms; flip angle: 3 degrees; receive bandwidth: 1290 Hz/pixel, that is, 454 kHz; encoded voxel dimensions and volume: 1.31 × 1.31 × 2.1 mm^3^; reconstructed voxel dimensions and volume: 0.65 × 0.65 × 2.00 mm^3^; slice oversampling: 40%; parallel‐imaging acceleration factor: 2; and superior and inferior spatial presaturation. Water signals only, fat signal only, in‐phase and opposed‐phase images were reconstructed, and the images of each type acquired at different stations were combined into a separate series for analysis.

### 2.3 Measurement and estimation of muscle volumes

**FIGURE 1:**
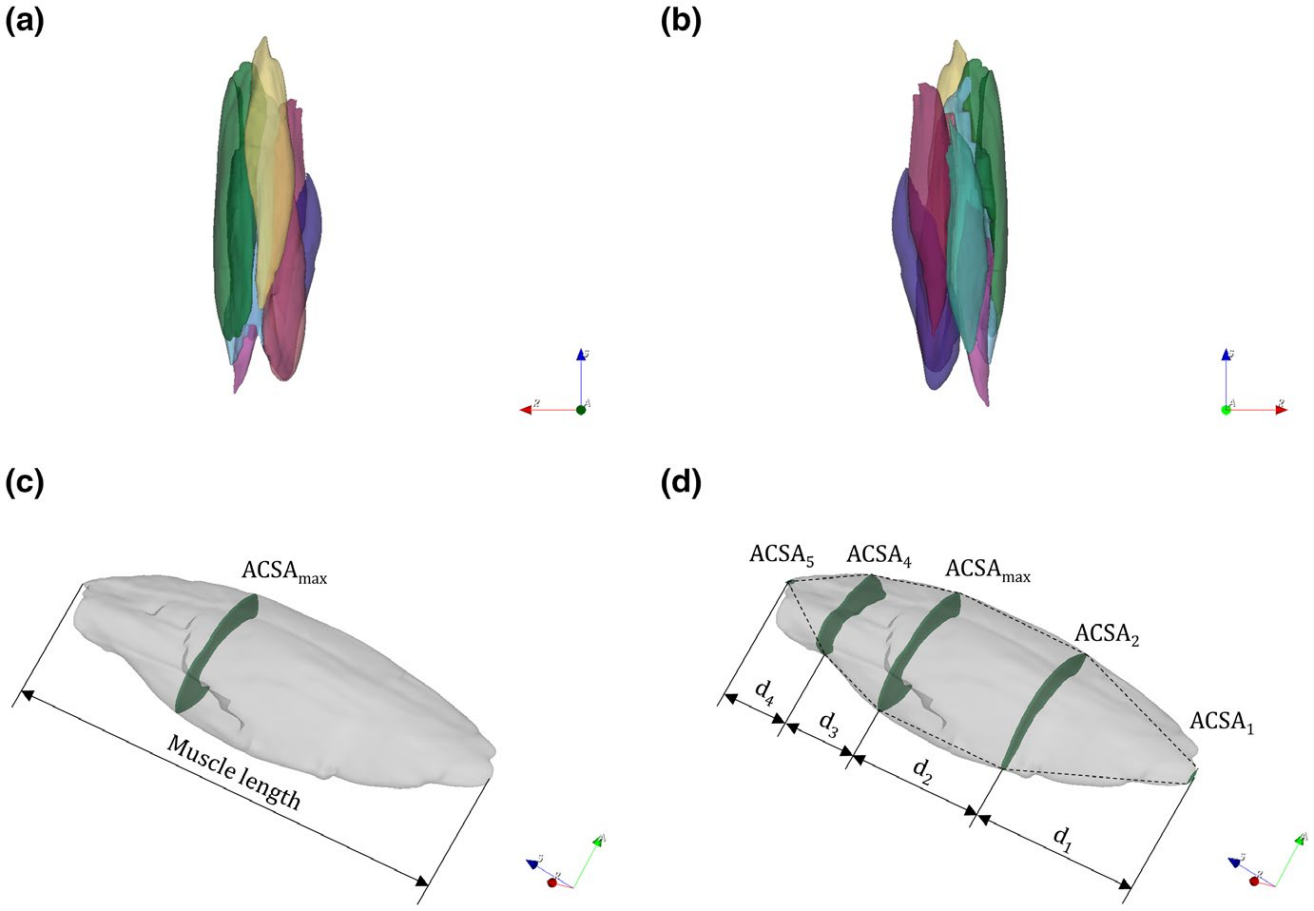
Examples of slice‐by‐slice segmented and reconstructed quadriceps and hamstring muscle volumes and graphical representations of the investigated estimation methods. (a) Anterior view. (b) Posterior view. (c) Shape factor method. (d) Truncated cone method. Green: Vastus lateralis, yellow: Rectus femoris, red: Vastus medialis, light blue: Vastus intermedius, pink: Biceps femoris short head, turquoise: Biceps femoris long head, burgundy red: Semitendinosus, purple: Semimembranosus.

MVs of the vastus lateralis (VL), rectus femoris (RF), vastus medialis (VM), vastus intermedius (VI), biceps femoris short head (BFsh), biceps femoris long head (BFlh), semitendinosus (ST), and semimembranosus of the participants' right thigh were obtained using the semiautomated and interactive open‐source MATLAB program SASHIMI Segmentation v1.2 (Bolsterlee, 2020). For this purpose, the ACSA was initially traced on a slice where the boundaries of the muscle were clearly visible (usually in the range of 50% of the muscle length). Based on this manually labeled ACSA, the program predicts the ACSA of the adjacent sections (either proximally or distally), which may require adjustment (i.e., move, add, or delete vertex). This procedure was repeated slice by slice (either proximally or distally) until the MVs were completely segmented from the point of insertion to the origin. Slice‐by‐slice segmentations were subsequently imported into the open‐source image computing platform 3D Slicer (Fedorov et al., 2012) to calculate the MVs using the Segment Statistics tool. Figure 1 illustrates an example of 3D volume reconstruction of the quadriceps and hamstring muscles from anterior (Figure 1a) and posterior (Figure 1b) perspectives.

3D Slicer built‐in extension SegmentGeometry (Huie et al., 2022) was used to determine ACSAs relative to muscle length (i.e., 1 ACSA value per 1% of muscle length). From the ACSAs normalized to the muscle length, the average location of the ACSA_max_ was identified in the next step. This site also formed the basis for the application of the two estimation methods. The estimation of MVs using the shape factor method (Figure 1c) was carried out in two steps. First, based on the theoretical assumption that the volume of a muscle equals a fraction of the product of muscle length and average ACSAmax (Albracht et al., 2008), muscle‐specific shape factors (*p*) were determined. For this purpose, the calculated MV from the slice‐by‐slice segmentation was divided by the product of muscle length and ACSA_max_ (Equation 1).

(1) $$p=\frac{{\mathrm{MV}}_{\text{slice}-\mathrm{by}-\text{slice}}}{\text{Muscle length}\times {\text{ACSA}}_{\max}}$$

Second, to estimate MVs, the product of the average muscle‐specific shape factor (*p*_avg_), muscle length, and average location slice of the ACSA_max_ was calculated (Equation 2).

(2) $$\begin{array}{c} {\mathrm{MV}}_{\text{shape factor}}=p_{\mathrm{avg}}\times \;\text{Muscle length}\\ \times \;\text{average location slice of}\;{\text{the ACSA}}_{\max} \end{array}$$

A total of 5 slices were used to estimate MVs via the truncated cone method (Figure 1d). In addition to the average location slice of the ACSA_max_, the slices at 1% (ACSA_5_) and 100% (ACSA_1_) of the muscle length, as well as 2 additional slices (ACSA_4_ and ACSA_2_) located halfway between ACSA_1_ and the average location slice of the ACSA_max_ and between the latter and ACSA_5_, respectively, were included in the calculation. Apart from the defined slices, the truncated cone method considers all distances between these slices and finally represents the sum of all partial volumes (Equation 3).

(3) $$\begin{array}{c} {\mathrm{MV}}_{\text{truncated cone}}=\sum_{n-1}\frac{d_{i}}{3}\times {\text{ACSA}}_{i}\\ +{\text{ACSA}}_{i+1}+\sqrt{{\text{ACSA}}_{i}\times {\text{ACSA}}_{i+1}} \end{array}$$

### 2.4 Statistical analysis

Statistical analyses and corresponding figure plotting were carried out using the statistical software GraphPad Prism 9.0.0 (Insight Partners, New York, United States). Descriptive data were expressed as the means ± standard deviations as a function of muscle length (from distal 0% to proximal 100%). To investigate the agreement between the shape factor method and the slice‐by‐slice method, and between the truncated cone method and the latter, a Bland–Altman analysis was performed for each quadriceps and hamstring muscle volume. Relative differences were plotted as a function of average values, the upper limit of agreement (ULOA) and lower limit of agreement (LLOA) were determined (±1.96 SD), and the mean difference (bias) was plotted.

## 3 RESULTS

An overview of the quadriceps and hamstring muscle parameters of female and male competitive soccer players, including MV_slice‐by‐slice_, muscle length, ACSA_max_, average location of ACSA_max_, and shape factor, can be found in Table S1.

### 3.1 Average location of the ACSA_max_ for quadriceps and hamstring muscles

**FIGURE 2:**
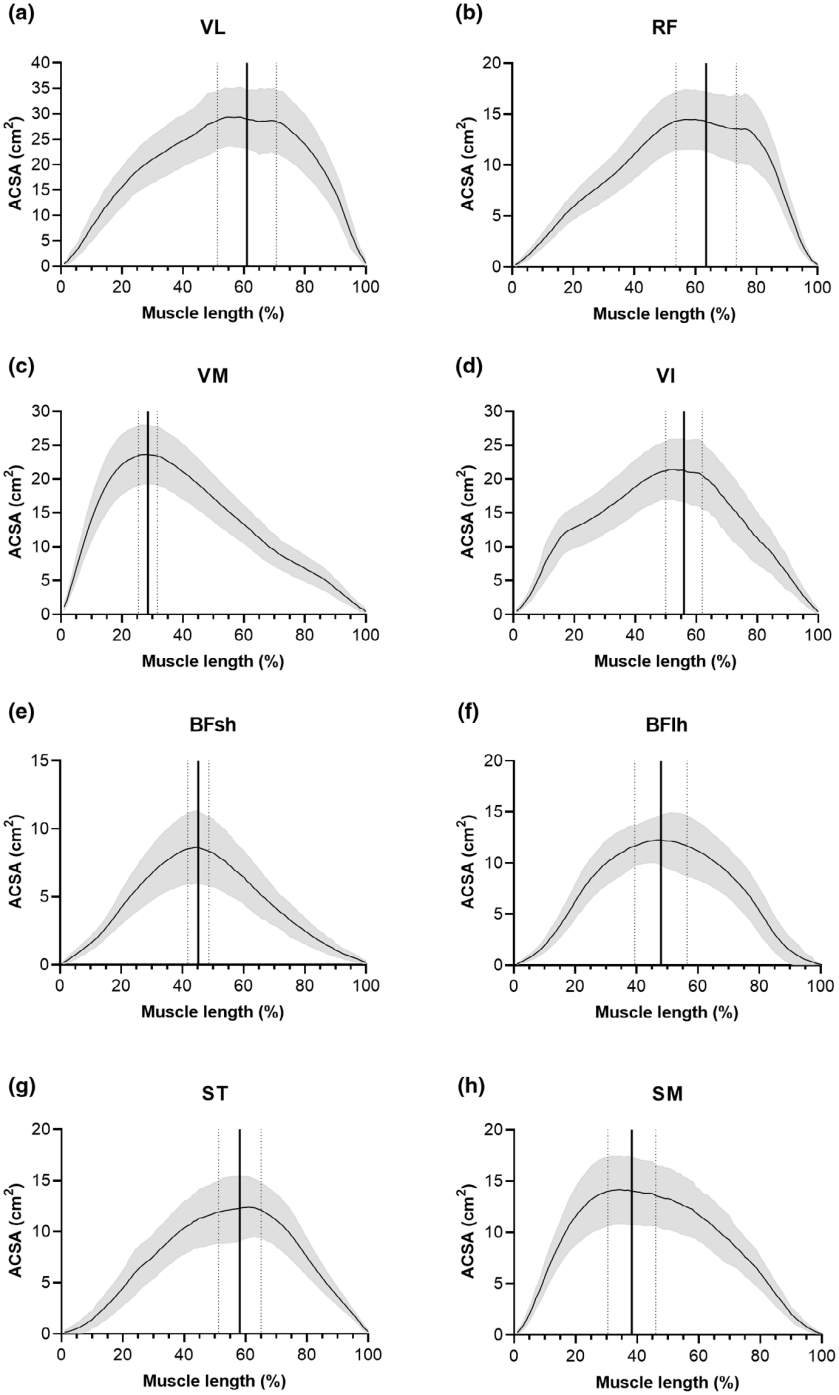
Average anatomical cross‐sectional area (ACSA) and standard deviation (gray area) of the quadriceps and hamstring muscles of soccer players (*n* = 39) as a function of relative muscle length (from distal 0% to proximal 100%). The vertical bold line indicates the average location of the maximal anatomical cross‐sectional area (ACSA_max_) (thin lines = standard deviation). BFlh, biceps femoris long head; BFsh, biceps femoris short head; RF, rectus femoris; SM, semimembranosus; ST, semitendinosus; VI, vastus intermedius; VL, vastus lateralis; VM, vastus medialis.

Figure 2 shows the average ACSA and standard deviation of the quadriceps (a–d) and hamstring (e, f) muscles as a function of muscle length (from distal 0% to proximal 100%) and the corresponding average location of the ACSA_max_ and standard deviation. For the quadriceps muscles, the average location of the ACSA_max_ was at 61.03 ± 9.65% for the VL, at 63.54 ± 9.90% for the RF, at 28.54 ± 3.12% for the VM, and at 55.99 ± 6.00% for the VI. For the hamstring muscles, the average location of the ACSA_max_ was at 45.18 ± 3.44% for the BFsh, at 47.95 ± 8.52% for the BFlh, at 58.15 ± 6.93% for the ST, and at 38.23 ± 7.78% for the SM.

### 3.2 Agreement between slice‐by‐slice segmentation and the shape factor method

**FIGURE 3:**
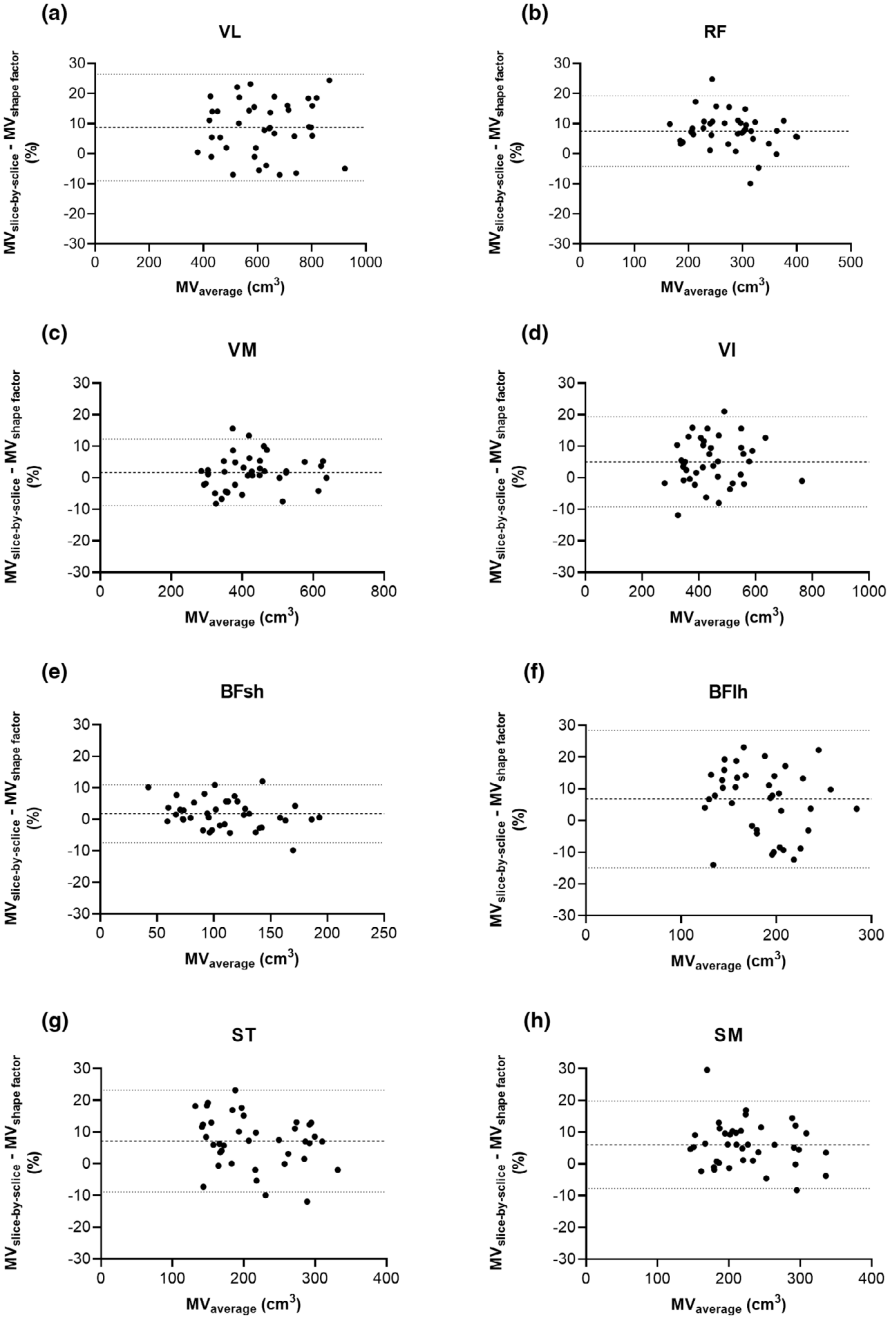
Bland–Altman analysis of the comparison between the slice‐by‐slice segmentation and the shape factor methods for the quadriceps and hamstring muscles of soccer players (*n* = 39). The horizontal bold dashed line represents the mean difference (bias), whereas the two thin dotted lines represent the upper limit of agreement (ULOA) and lower limit of agreement (LLOA). BFlh, biceps femoris long head; BFsh, biceps femoris short head; MV, muscle volume; RF, rectus femoris; SM, semimembranosus; ST, semitendinosus; VI, vastus intermedius; VL, vastus lateralis; VM, vastus medialis.

Figure 3 shows the relative average differences (biases) and the lower and upper limits of agreement (LLOA and ULOA, respectively) between the slice‐by‐slice segmentation and the shape factor methods for the quadriceps (a‐d) and hamstring (e‐f) MVs. For the quadriceps MVs, Bland–Altman analysis revealed a relative bias of 8.76 ± 9.04% (LLOA: −8.96%, ULOA: 26.5%) for the VL, of 7.54 ± 5.98% (LLOA: −4.18%, ULOA: 19.20%) for the RF, of 1.68 ± 5.38% (LLOA: −8.87%, ULOA: 12.20%) for the VM, and for the VI of 5.04 ± 7.30% (LLOA: −9.27%, ULOA: 19.30%). For hamstring MVs, Bland–Altman analysis revealed a relative bias of 1.76 ± 4.67% (LLOA: −7.40%, ULOA: 10.9%) for the BFsh, of 6.77 ± 11.10% (LLOA: −14.90%, ULOA: 28.50%) for the BFlh, of 7.14 ± 8.20% (LLOA: −8.93%, ULOA: 23.20%) for the ST, and for the SM of 6.02 ± 7.05% (LLOA: −7.80%, ULOA: 19.80%).

### 3.3 Agreement between slice‐by‐slice segmentation and the truncated cone method

**FIGURE 4:**
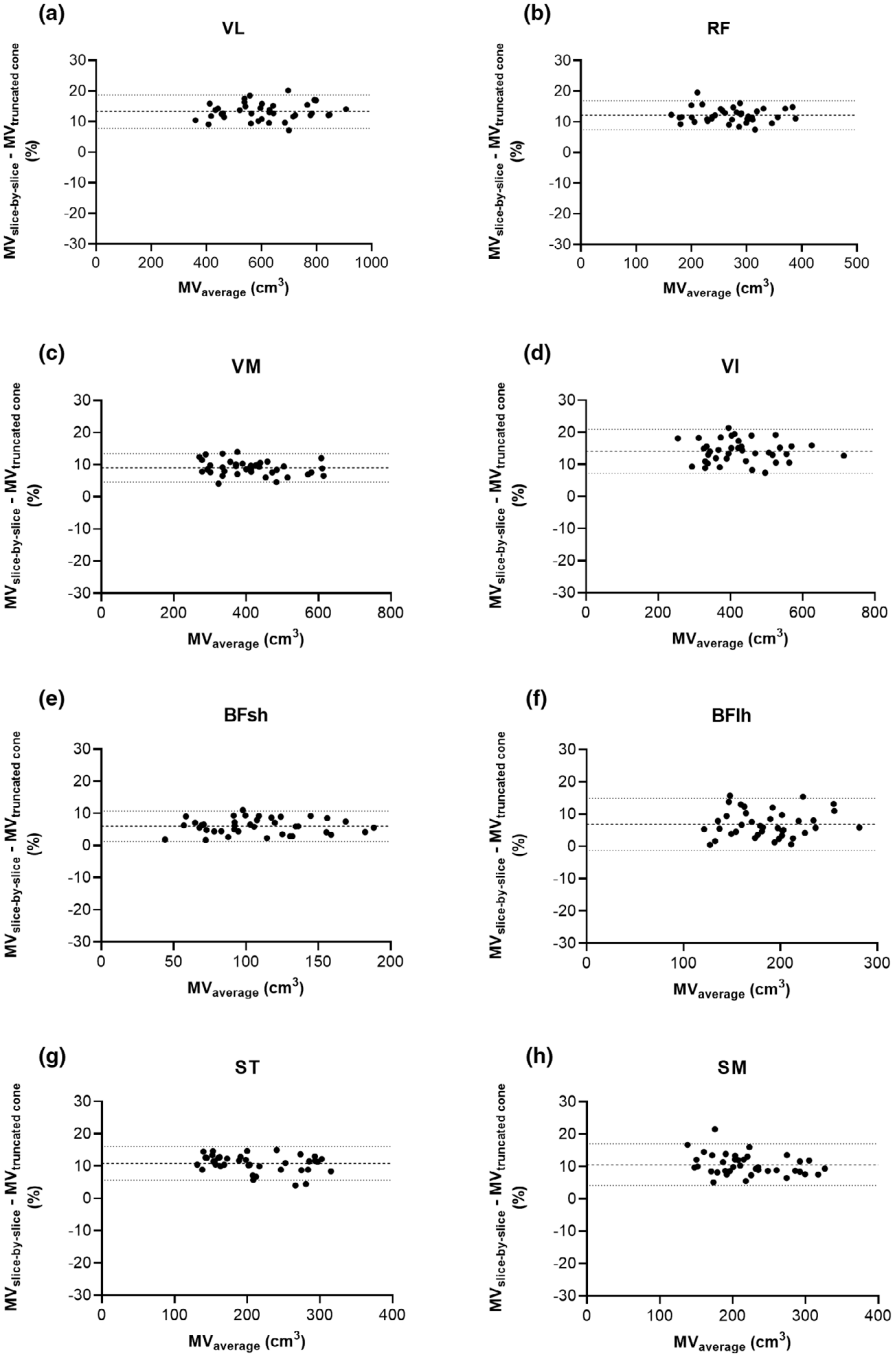
Bland–Altman analysis of the comparisons between the slice‐by‐slice segmentation and the truncated cone methods for the quadriceps and hamstring muscles of soccer players (*n* = 39). The horizontal bold dashed line represents the mean difference (bias), whereas the two thin dotted lines represent the upper limit of agreement (ULOA) and lower limit of agreement (LLOA). BFlh, biceps femoris long head; BFsh, biceps femoris short head; MV, muscle volume; RF, rectus femoris; SM, semimembranosus; ST, semitendinosus; VI, vastus intermedius; VL, vastus lateralis; VM, vastus medialis.

Figure 4 shows the relative average differences (biases) and the lower and upper limits of agreement (LLOA and ULOA, respectively) between the slice‐by‐slice segmentation and the truncated cone methods for the quadriceps (a–d) and hamstring (e, f) MVs. For the quadriceps MVs, Bland–Altman analysis revealed a relative bias of 13.30 ± 2.78% (LLOA: 7.83%, ULOA: 18.70%) for the VL, of 12.10 ± 2.39% (LLOA: 7.45%, ULOA: 16.80%) for the RF, of 9.04 ± 2.27% (LLOA: 4.58%, ULOA: 13.50%) for the VM, and for the VI of 14.10 ± 3.48% (LLOA: 7.25%, ULOA: 20.90%). For hamstring MVs, Bland–Altman analysis revealed a relative bias of 5.97 ± 2.41% (LLOA: 1.26%, ULOA: 10.70%) for the BFsh, of 6.87 ± 4.12% (LLOA: −1.21%, ULOA: 14.90%) for the BFlh, of 10.80 ± 2.68% (LLOA: 5.57%, ULOA: 16.10%) for the ST, and for the SM of 10.50 ± 3.28% (LLOA: 4.11%, ULOA: 17.00%).

## 4 DISCUSSION

The present study provided reference values for the average locations of the ACSA_max_ (relative to muscle length) for the individual quadriceps and hamstring muscles. Additionally, the accuracy and precision of the shape factor and the truncated cone method were compared to quantify the quadriceps and hamstring MVs. While the shape factor method displayed better accuracy (i.e., smaller average differences, bias), the truncated cone method underestimated MVs on average to a greater extent but with better precision (i.e., the range between the LLOA and ULOA was smaller).

While the anatomical locations of the ACSA_max_ for the VL, RF, VI, and ST were in the range of 60% of muscle length (i.e., more towards proximal), they were close to 50% for the BFsh and BFlh and 30% and 40% for the VM and SM, respectively (i.e., more towards distal). These results could serve as a reference if (for time and cost reasons) only a single ACSA measurement can be performed (e.g., with a US system) or as a starting point for future practical implementations of the shape factor or truncated cone method. Direct comparison of the average location of the ACSA_max_ to the literature proves difficult because most of the studies (Balshaw et al., 2021; Behan et al., 2018; Morse et al., 2007) have determined this site relative to femur length. It should also be considered that in some studies (Balshaw et al., 2021; Behan et al., 2018; Morse et al., 2007), 0% corresponded to the distal end and 100% to the proximal end, whereas in another study, it was vice versa (Mersmann et al., 2015). Nevertheless, a visual comparison of the curves of the average ACSA revealed very similar profiles to those of previous studies for the quadriceps and hamstring muscles (Balshaw et al., 2021; Behan et al., 2018; Mersmann et al., 2015; Morse et al., 2007).

The muscle‐specific shape factors for the VL (0.62), the VM (0.55), and the VI (0.59) were comparable to those reported by Mersmann et al. (2015) (VL: 0.65, VM: 0.54, and VI: 0.58), who studied an athlete cohort of 37 female and male volleyball players. In the study by Domroes et al. (2021), 24 trained and untrained early‐adolescent handball and basketball athletes aged 12–14 years also resulted in a shape factor of 0.62 for the VL. Thus, the present study provides further evidence that muscle‐specific shape factors for those quadriceps muscles are relatively constant across a population. The shape factors of the RF and hamstring muscles were, to the best of our knowledge, collected for the first time within the present study and can be found in Table S1. Furthermore, it is important to emphasize that no sex differences were found in either the quadriceps or the hamstring regarding the shape factor, further suggesting that muscle‐specific shape factors are not dependent on sex.

Examining the agreement between the slice‐by‐slice segmentation and the shape factor methods revealed that the bias was smaller for muscles in which the average location of the ACSA_max_ displayed a smaller standard deviation (i.e., VM and BFsh). Across all quadriceps and hamstring muscles, the bias was in the range of less than 10%. However, the scatter of data points was quite large, especially for the VL and BFlh, resulting in a large range between the LLOA and the ULOA. Importantly, these results are derived from a variant of the shape factor method that is directly applicable in practice; if the effective ACSA_max_ (see Table S1) was considered for muscle volume calculation, the biases would be very close to zero and smaller ranges between the LLOA and ULOA would result. For these reasons, the shape factor method would thus be appropriate when the average values of a sample of muscle volumes are to be examined (e.g., for a cross‐sectional study) in a time‐efficient manner. Direct comparisons of bias, LLOA, and ULOA to the literature cannot be drawn because previous studies (Albracht et al., 2008; Domroes et al., 2021; Karamanidis et al., 2019; Mersmann et al., 2014, 2015) did not perform Bland–Altman analyses.

Analysis of the agreement between the slice‐by‐slice segmentation and the truncated cone methods revealed larger biases than for the shape factor method, but the scatter of the individual data points was smaller (i.e., a smaller range between the LLOA and ULOA). In other words, muscle volumes were systematically underestimated with the truncated cone method but with better precision. Previous studies (Lund et al., 2002; Nordez et al., 2009; Tracy et al., 2003) have shown that the agreement of the truncated cone method improved with an increasing number of segmented slices or shorter interslice intervals. These studies also documented an underestimation of muscle volume using the truncated cone method. Nordez et al. (2009) found comparable biases (in the range of 10%) for the quadriceps muscles for the same number of segmented slices.

While interpreting the results of the present study, it is important to consider that the study included only competitive soccer players (i.e., a population with specific training‐induced adaptations). This likely limits the generalizability of the results to other populations, such as untrained athletes, athletes from other sports, or clinical populations. Further studies are needed to determine whether the location of the ACSA_max_ and the agreement between the methods remain constant in diverse cohorts.

Although it may seem counterintuitive to use the gold standard (i.e., MRI) for muscle imaging and then apply estimation methods, in our view there are cases where slice‐by‐slice segmentation is not practical enough due to time constraints. In our experience, even semi‐automatic slice‐by‐slice segmentation of a leg with 8 muscles takes about 3–4 h. Using the shape factor method, we estimate about 40 min (5 min per muscle) and using the truncated cone method about 1 h 20 min (10 min per muscle) for the same work. Therefore, these alternative estimation methods are not intended to replace the slice‐by‐slice approach, but they could be used if the results need to be obtained in a timely manner (e.g., as soon as possible after the test) and the sample size is relatively large (e.g., many athletes tested on the same day). Another advantage of both estimation methods is that they could also be applied with a US system. In order to determine the measurement locations relative to the muscle length, the distal and proximal muscle‐tendon junctions would have to be palpated or identified with the US transducer, and the measurement locations determined using a measuring tape. The measurements of the ACSAs would then be carried out using transversal panoramic mode US scans.

## 5 CONCLUSIONS

The results of the present study illustrate that the quadriceps and hamstring muscles vary in terms of the anatomical location of the ACSA_max_ (relative to muscle length). Accordingly, our results could serve as a reference if (for time and cost reasons) only a single ACSA measurement can be performed (e.g., with a US system). When comparing slice‐by‐slice segmentation (reference standard) with the shape factor method, the mean differences were smaller than those of the truncated cone method. Conversely, the shape factor method resulted in greater scatter of individual data, which affected the range between the LLOA and ULOA. Thus, based on the required accuracy and/or precision, a choice could be made between the two estimation methods investigated in this study in sports and clinical practice. While the estimation of muscle volume using the shape factor method may be more suitable for the time‐efficient analysis of MR images to determine the mean value of a sample in a cross‐sectional study, the truncated cone method may be better suited for the measurement of individual trajectories during a longitudinal study design and could also be accomplished with a US system.

## FUNDING INFORMATION

The study was funded internally by the Balgrist Foundation.

## CONFLICT OF INTEREST STATEMENT

The authors disclose no conflicts of interest.

## ETHICS APPROVAL STATEMENT

The underlying study protocol was approved by the Cantonal Ethics Committee Zurich (BASEC No. 2020–02583), and the study was conducted according to the ethical standards of the Declaration of Helsinki and national laws. All study participants signed a study‐specific informed consent form before participation.

## Supporting information


Table S1.

## Data Availability

The datasets generated and/or analyzed for the current study are not directly publicly available but are available on reasonable request from J.S. (joerg.spoerri@balgrist.ch).
