# Supplementary material for: Shape factor versus truncated cone‐based quantification of quadriceps and hamstring muscle volumes—A choice between accuracy and precision
Source: Physiol Rep. 2025 Mar 25;13(6):e70263. doi: 10.14814/phy2.70263 (PMC11933715; doi:10.14814/phy2.70263)
Supplement: Supplementary file 1 — Table S1. [file PHY2-13-e70263-s001.docx]

# Supplementary material

Table 1: Overview of quadriceps and hamstring muscle parameters of female and male competitive soccer players (n = 39).

| Parameter | Main  effects | Muscle | Overall  (n = 39) | Female  (n = 19) | Male  (n = 20) |
| --- | --- | --- | --- | --- | --- |
| MV_slice-by-slice_ (cm^3^) | * # × | VL | 648.55 ± 150.26 | 534.40 ± 90.57 | 756.99 ± 109.98 |
|  |  | RF | 287.80 ± 61.01 | 244.52 ± 42.33 | 328.91 ± 45.68 |
|  |  | VM | 430.10 ± 99.44 | 363.49 ± 60.80 | 493.38 ± 86.91 |
|  |  | VI | 459.27 ± 104.33 | 382.07 ± 52.77 | 532.61 ± 86.84 |
|  |  | BFsh | 110.94 ± 36.46 | 81.87 ± 17.33 | 138.55 ± 27.23 |
|  |  | BFlh | 191.99 ± 39.65 | 165.78 ± 25.89 | 216.90 ± 34.00 |
|  |  | ST | 222.03 ± 58.92 | 175.43 ± 25.90 | 266.30 ± 45.93 |
|  |  | SM | 231.37 ± 52.07 | 194.39 ± 28.04 | 266.49 ± 44.80 |
| Muscle length (cm) | * # | VL | 33.10 ± 2.32 | 31.84 ± 2.41 | 34.29 ± 1.42 |
|  |  | RF | 32.16 ± 2.03 | 31.40 ± 1.77 | 32.89 ± 2.00 |
|  |  | VM | 32.17 ± 2.20 | 31.22 ± 1.68 | 33.08 ± 2.25 |
|  |  | VI | 34.90 ± 1.89 | 34.09 ± 1.55 | 35.67 ± 1.86 |
|  |  | BFsh | 25.23 ± 1.89 | 24.65 ± 0.50 | 25.78 ± 1.70 |
|  |  | BFlh | 27.67 ± 2.17 | 26.95 ± 1.92 | 28.36 ± 2.16 |
|  |  | ST | 31.09 ± 2.05 | 30.24 ± 2.05 | 31.89 ± 1.69 |
|  |  | SM | 26.37 ± 2.48 | 25.52 ± 2.32 | 27.18 ± 2.35 |
| ACSA_max_ (cm^2^) | * # × | VL | 31.75 ± 6.47 | 27.09 ± 3.58 | 36.17 ± 5.41 |
|  |  | RF | 15.32 ± 2.99 | 13.57 ± 2.22 | 16.99 ± 2.65 |
|  |  | VM | 24.02 ± 4.37 | 21.40 ± 3.01 | 26.50 ± 3.99 |
|  |  | VI | 22.42 ± 4.55 | 19.34 ± 1.65 | 25.33 ± 4.51 |
|  |  | BFsh | 8.78 ± 2.64 | 6.70 ± 1.27 | 10.77 ± 2.00 |
|  |  | BFlh | 13.18 ± 2.69 | 11.89 ± 2.11 | 14.41 ± 2.20 |
|  |  | ST | 13.18 ± 3.17 | 10.74 ± 1.68 | 15.49 ± 2.44 |
|  |  | SM | 14.87 ± 3.26 | 12.97 ± 1.66 | 16.68 ± 3.37 |

| Average location of ACSA_max_ (%) | # | VL | 61.03 ± 9.65 | 60.00 ± 9.12 | 62.00 ± 10.03 |
| --- | --- | --- | --- | --- | --- |
|  |  | RF | 63.54 ± 9.90 | 63.42 ± 9.10 | 63.65 ± 10.60 |
|  |  | VM | 28.54 ± 3.12 | 27.79 ± 2.44 | 29.25 ± 3.51 |
|  |  | VI | 55.99 ± 6.00 | 54.24 ± 5.07 | 57.65 ± 6.33 |
|  |  | BFsh | 45.18 ± 3.44 | 45.15 ± 3.79 | 45.20 ± 3.08 |
|  |  | BFlh | 47.95 ± 8.52 | 47.11 ± 7.09 | 48.75 ± 9.62 |
|  |  | ST | 58.15 ± 6.93 | 59.95 ± 7.44 | 56.45 ± 5.93 |
|  |  | SM | 38.23 ± 7.78 | 39.58 ± 6.39 | 36.95 ± 8.70 |
| Shape factor | # | VL | 0.62 ± 0.04 | 0.62 ± 0.04 | 0.61 ± 0.04 |
|  |  | RF | 0.58 ± 0.04 | 0.58 ± 0.04 | 0.59 ± 0.02 |
|  |  | VM | 0.55 ± 0.03 | 0.54 ± 0.03 | 0.56 ± 0.03 |
|  |  | VI | 0.59 ± 0.03 | 0.58 ± 0.03 | 0.59 ± 0.04 |
|  |  | BFsh | 0.50 ± 0.02 | 0.50 ± 0.02 | 0.50 ± 0.02 |
|  |  | BFlh | 0.53 ± 0.05 | 0.52 ± 0.04 | 0.54 ± 0.05 |
|  |  | ST | 0.54 ± 0.04 | 0.54 ± 0.05 | 0.54 ± 0.03 |
|  |  | SM | 0.59 ± 0.03 | 0.59 ± 0.02 | 0.59 ± 0.03 |

Data are expressed as the mean ± standard deviation. Statistics: Two-way analyses of variance with fixed factors sex and muscle. Main effects: * = significant effect of sex, # = significant effect muscle, x = significant interaction effect between sex and muscle. P < 0.05. MV = muscle volume, ACSA_max_ = maximal anatomical cross-sectional area, VL = vastus lateralis, RF = rectus femoris, VM = vastus medialis, VI = vastus intermedius, BFsh = biceps femoris short head, BFlh = biceps femoris long head, ST = semitendinosus, SM = semimembranosus.
